# Microalgal Microscale Model for Microalgal Growth Inhibition Evaluation of Marine Natural Products

**DOI:** 10.1038/s41598-018-28980-z

**Published:** 2018-07-12

**Authors:** Qing Zhao, An-Na Chen, Shun-Xin Hu, Qian Liu, Min Chen, Lu Liu, Chang-Lun Shao, Xue-Xi Tang, Chang-Yun Wang

**Affiliations:** 1 Key Laboratory of Marine Drugs, the Ministry of Education of China, School of Medicine and Pharmacy, Ocean University of China, Qingdao, 266003 China; 2 Laboratory for Marine Drugs and Bioproducts, Qingdao National Laboratory for Marine Science and Technology, Qingdao, 266071 P. R. China; 3 College of Marine Life Sciences, Ocean University of China, Qingdao, 266003 China; 4 Marine Science & Technology Institute, College of Environmental Science & Engineering, Yangzhou University, 196#, Huayang West Street, Yangzhou, 225127 China; 5 Institute of Evolution & Marine Biodiversity, Ocean University of China, Qingdao, 266003 China

## Abstract

Marine organisms especially sessile invertebrates, such as soft corals, gorgonians and sponges, can survive in the competitive environment mainly relying on their second metabolites with chemoecological effects including allelopathy and algal growth inhibition. It is well known that the microscale models are urgently needed in marine chemoecology assessment to evaluate the algal growth inhibition activity of trace quantity natural products. In this work, a microalgal growth inhibition model was established for microalgal inhibition evaluation of marine natural products with 96-well microplate by automatic fluorescence observation using microplate reader. Subsequently, this model was applied to bioassay-guided isolation and preliminary bioactivity screening of the secondary metabolites from soft corals, gorgonians, sponges and their symbiotic microbes collected from the South China Sea. As a result, fifteen compounds (**1**‒**15**) were found to exhibit microalgal growth inhibition activities against at least one of marine microalgae, *Karenia mikimotoi*, *Isochrysis galbana*, and *Heterosigma akashiwo*. Specifically, altersolanol C (**13**) demonstrated potent activity against *K. mikimotoi* with the 96h-EC_50_ value of 1.16 µg/mL, more than four times stronger than that of the positive control K_2_Cr_2_O_7_. It was suggested that the microalgal growth inhibition microscale model is suitable for bioassay-guided isolation and preliminary bioactivity screening of marine natural products.

## Introduction

In marine ecosystem, marine organisms especially sessile invertebrates, such as soft corals, gorgonians and sponges, can survive in the competitive environment mainly relying on their second metabolites with chemical ecology functions. The secondary metabolites from marine invertebrates and their symbiotic microbes have been found to serve as defensive and allelopathic substances possessing various chemical ecology effects, such as algal growth inhibition, antifouling, allelopathy, ichthyotoxicity, insecticidal, antipredatory and antimicrobial activities^1–7^. To evaluate the chemoecological effects of secondary metabolites isolated from marine organisms, many models have been established and applied in the field of marine chemical ecology^3,5,6^. It has been concerned that the microscale models for marine chemoecology assessment are urgently needed for trace quantity of marine natural products.

Since 1971, microalgal models have been used to test the microalgal growth-inhibition activities^8–12^. Marine microalgae, as the dominant primary producers, are considered as an ecologically important group playing an essential role in the marine ecosystem^13^. Marine microalgae have been testified as the basic link in aquatic food chains and a key functional group of marine organisms^14^. In recent years microalgal blooms have caused serious problems in eutrophic water bodies by destroying the ecological balance^15,16^. The public health and economic problems caused by harmful algal blooms appear to have increased in frequency, intensity, and geographic distribution over the past decades^17^. Microalgae were reported to have greater sensitivity than invertebrates and fish to natural products as well as municipal and industrial effluents^18–24^. Based on these facts, microalgae have been widely used in the microalgal models to estimate the ecotoxicological character of environmental samples. Specifically, microalgal models have been used as a microscale model in the fields of environment ecological evaluation, including herbicides, pestcides, industrial wastes and sediment, as well as the heavy metal toxicity assessment in marine and aquatic systems^10,25–29^.

Traditionally, the classic models for microalgal growth-inhibitory activity were conducted with conical glass flasks, and a large test volume, e.g. 50 mL to 500 mL, is normally used in practical experiments^30^. In order to improve the efficiency of standard test procedures, the test vessels were necessary to be miniaturized, and the miniature methods with 96-well microplates (250 μL/well) were proposed and applied^10^. The traditional 250 mL Erlenmeyer flask vessels were substituted by microplates requiring only 2 mL or less test volume^10,31,32^. There have been several approaches to develop a small-scale microplate toxicity test, together with the ways of quantifying microalgal growth such as cell counting with electronic particle counting optical density (O.D.) measurement^31–34^, fluorescence measurement^26,35^, and ATP quantification^36^.

It should be noted that the microalgal microscale models have been seldom reported for the chemoecological assessment of marine natural products. Due to the fact that marine natural products exist in the original organisms with usually micro- or trace-scale (ppm, or even ppb), it is necessary to build a microscale model for microalgal inhibition evaluation. Based on the above consideration, in the present study, we modified a microalgal growth inhibition microscale model by using 96-well microplates with three microalgae and measuring the chlorophyll fluorescence based on microalgal biomass. This improved model would be suitable for the assessment of the microalgal inhibition activities, specifically for micro- or trace marine natural products. The model was applied to microalgal inhibition activity assessment for the secondary metabolites isolated from corals, gorgonians, sponges and their symbiotic microorganisms. Herein, we report how to evaluate the microalgal inhibition activity of the micro- or trace marine natural products in a convenient and efficient way.

## Materials and Methods

### General experimental procedures

Microplate reader SpetraMax M5 (Molecular Instrument Company, USA) was used to estimate the microalgal biomass through chlorophyll fluorescence measurement^26,35^. Inverted fluorescence microscope DMI 6000B (Leica Microsystems Company, Germany) was used for the observation of microalgal fluorescence. 96-Well microplates (Beijing Siqi Biological Technology Company, China) were used for the microalgal growth inhibition tests. Intelligent illumination incubator GXZ (Ningbo Jiangnan Park New Science and Technology Company, China) was used for cultivation of marine microalgae. Potassium dichromate (K_2_Cr_2_O_7_) (Tianjin Guangcheng Chemical Company, China, purity ≥99.9%) was used as a reference substance and a positive control. Dimethyl sulfoxide (DMSO) (Tianjin Guancheng Chemical Reagent Company, China, purity ≥99.9%) was used as cosolvent to improve the solubility of the tested compounds. Considering of the dissolvability for the most of tested marine natural products and almost no observed inhibitory effect on microalgae, 0.5% DMSO was chosen as the cosolvent and negative control. The nutritive medium composed of natural seawater and a supply of nutrients and vitamins according to the f/2 (half strength f medium) nutritive medium^37^. Natural seawater obtained from Qingdao offshore was used to configure the f/2 culture medium after using 0.45 μm microporous membrane filter.

### Microalgal species and culture condition selection

The marine microalgal species *Karenia mikimotoi*, *Isochrysis galbana* and *Heterosigma akashiwo* were supplied by the Laboratory of Ecological, Ocean University of China. These microalgae were maintained and precultured according to ISO 8692^38^, with f/2 medium kept at 25 °C, pH 8.0 ± 0.1 under a 12: 12 light: dark cycle using a photon flux density of 80 μmol photon/m^2^·s and were shaken three times daily. In order to determine the exponential growth phases of three microalgae, we investigated the growth patterns of these three microalgae in 250 mL Erlenmeyer flasks with three replicates (*n* = 3). The initial concentrations of *K. mikimotoi*, *I. galbana*, and *H. akashiwo* were 5 × 10^4^ cells/mL, 1 × 10^5^ cells/mL, and 5 × 10^4^ cells/mL, respectively.

### Chlorophyll fluorescence value measurement

Microplate reader SpectraMax M5 was used to acquire the fluorometric data, with excitation wavelength of 485 nm and emission wavelength of 680 nm. As transparent polystyrene microplates cannot be used for fluorometric measurement, 96-well nontransparent polystyrene microplates with transparent bottom were used. At the time point of 96 h, the microalgal culture was transported from 96-well transparent microplates to 96-well nontransparent polystyrene microplates with transparent bottom. Then the fluorometric data were recorded using microplate reader. The 96 h half maximal effective concentration (96h-EC_50_) value of each compound was estimated with different concentrations at 96 h^10^.

### Statistical ananlysis

The intensity fluorescence values were obtained from microplate reader. These data were first processed by Excel, the initial and endpoint measurement were used to calculate the percentage of microalgal growth inhibition corresponding each concentration of tested substances. Then the 96h-EC_50_ values were determined by applying a PROBIT analysis^39^ with the aid of the statistical software SPSS 17.0.

### Microalgal growth inhibition bioassay

**Figure 1:**
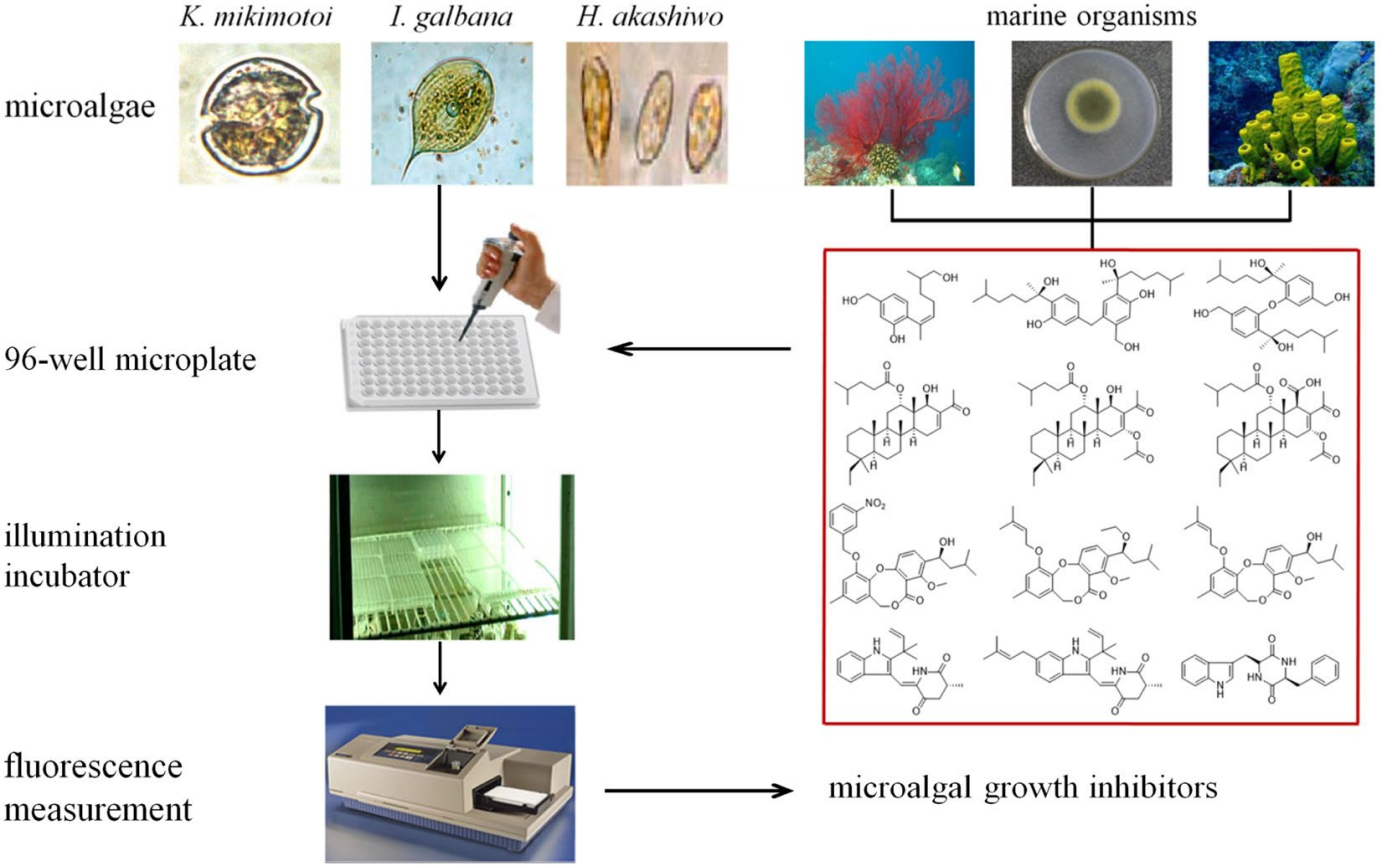
The main procedure of microalgal growth inhibition assay for the assessment of marine natural products.

The procedures of the bioassay could be summarized as follows. In order to reduce evaporation, sterile water was added to the exterior ring wells of 96-well transparent microplate. Precultured exponentially-growing microalgae of *K. mikimotoi* (6th day, 5 × 10^4^ cells/mL), *I. galbana* (21st day, 1 × 10^5^ cells/mL), and *H. akashiwo* (15th day, 5 × 10^4^ cells/mL) were moved to the wells of 96-well transparent microplate using a pipette. Each well was injected with 199 μL of microalgal suspension and 1 μL of test solution containing the tested compound with different concentration prepared in advance to make a final volume of 200 μL. To measure the microalgal growth inhibition activity, the maximum amount required for each compound was 200 μg. The tested compounds were dissolved in 0.5% (v/v) DMSO firstly^30^, and then diluted with 0.5% DMSO to obtain five test concentrations. The final concentrations of the tested compounds in each well of 200 μL culture system were 50 μg/mL, 10 μg/mL, 2 μg/mL, 0.4 μg/mL, and 0.08 μg/mL, respectively. For the blank control, positive control and negative control, 1 μL H_2_O, 1 μL K_2_Cr_2_O_7_ (2 μg/mL) and 1 μL 0.5% DMSO were added into microalgal suspensions instead of the test compound solutions, respectively. Each sample was tested in three replicates. The 96-well transparent microplates were incubated at 25 °C, pH 8.0 ± 0.1 under a 12 h light/12 h dark photoperiod using a photon flux density of 80 μmol photon/m^2^·s and were shaken thrice daily. In the course of the whole experiment, microalgal culture conditions remained consistent. The fluorometric values reflecting microalgal biomass were measured by microplate reader. The measured initial and endpoint fluorescence values representing the microalgal biomass were used to calculate the percentage of growth inhibition, then the 96h-EC_50_ values were calculated. The above procedures were summarized in Fig. 1.

In order to investigate the reproducibility and feasibility of the established microplate microalgal biotest model, three repeated tests were conducted with six replicates per concentration of the positive control K_2_Cr_2_O_7_. The final concentrations of positive control K_2_Cr_2_O_7_ in each well of 96-well microplate (200 μL) were 16, 8, 4, 2, 1, and 0.5 μg/mL, respectively.

### Bioassay-guided isolation for secondary metabolites from marine organisms

The secondary metabolites were isolated under the guidance of microalgal microscale model from marine organisms, including corals, gorgonians, sponges and their symbiotic microorganisms collected from the South China Sea. To assess the microalgal growth inhibition activity, a maximum amount of 100 µg for each extract or fraction was required. All of the compounds were isolated by using chromatographic techniques including column chromatography and semi-preparative HPLC. Taking a soft coral-derived fungus, *Alternaria* sp., as an example, the procedures of the bioassay-guided isolation for secondary metabolites from this fungus were as follows. The ethyl acetate extract of the fungal fermentation broth was tested for its microalgal growth inhibition activity. The active extract was then subjected to silica gel column chromatography using gradient elution with petroleum ether/ethyl acetate mixtures of increasing polarity. The fractions and subfractions were obtained and tested for their microalgal growth inhibition activities. Trough further bioassay-guided isolation by column chromatography and semi-preparative HPLC, pure compounds were obtained eventually. The similar approaches of bioassay-guided isolation were applied to the isolation of the secondary metabolites from other marine species. The structures of all the isolated compounds were elucidated by comprehensive analysis of spectroscopic data, including IR, UV, NMR, MS, and X-ray^40–42^.

## Result and Discussions

### Establishment of microalgal growth inhibition microscale model

**Figure 2:**
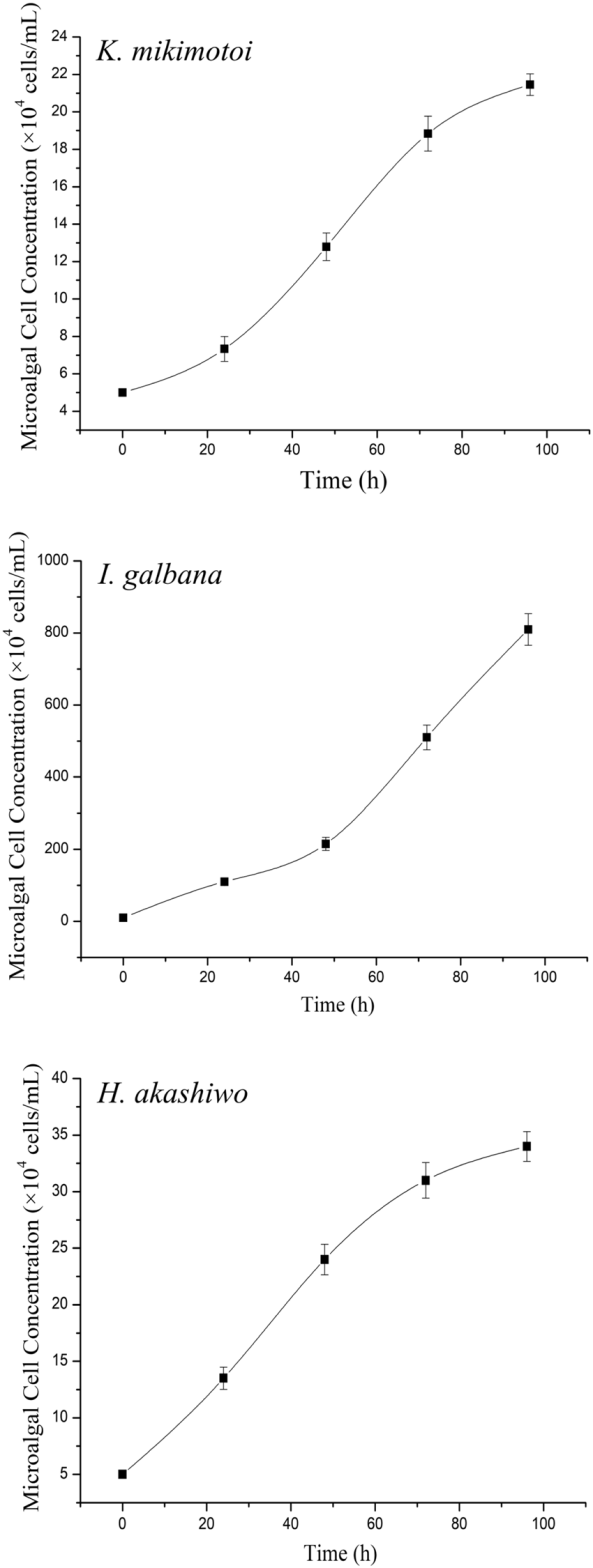
Growth curves for populations of three microalgae during 96 hours in 96-well microplates (*n* = 3).

In this study, the microalgal growth inhibition model was applied based on microalgal biomass. Different from the previous reported microalgal model with 96-well microplates^32^, the model in present study was operated with three marine microalgal species, *K. mikimotoi*, *I. galbana* and *H. akashiwo* because they are sensitive to tested substances and could be isolated and cultured easily in laboratory. *K. mikimotoi* and *H. akashiwo* were harmful red tide algae, while *I. galbana* was frequently used in microalgal growth inhibition tests as a standard microalga^11^. Through pre-experiments, the exponential growth phases of three microalgae were found to be in the periods of 5‒27, 3‒25, and 3‒23 days, respectively (see Supplementary Fig. S1). Subsequently, the growth curves for populations of these three microalgae in 96-well transparent microplates during 96 hours were inspected. During the period of 96 h, three microalgae grew with a huge variation and *I. galbana* grew fastest among these three microalgae (Fig. 2). It has been revealed that the tested sensitivity increased with decreasing initial microalgal cellular concentration^43–45^. Considering the maximum sensitivity of the bioassay and sufficient cells to determine cellular concentration changes over the test duration, most test protocols recommended an initial cellular concentration of 10^4^ to 10^5^ cells/mL^46^. In our study, the initial concentration of *K. mikimotoi* and *H. akashiwo* was chosen as 5 × 10^4^ cells/mL, while that of *I. galbana* was twice. Based on the exponential growth phases, we choose the three microalgae at 6th day for *K. mikimotoi*, 21st day for *I. galbana*, and 15th day for *H. akashiwo*, to ensure the microalgal cells in exponential growth status and good cellular viability. Then, for the bioassay, we diluted and inoculated the microalgae to the wells of 96-well transparent microplate separately according to the designed initial microalgal cellular concentration above.

**Figure 3:**
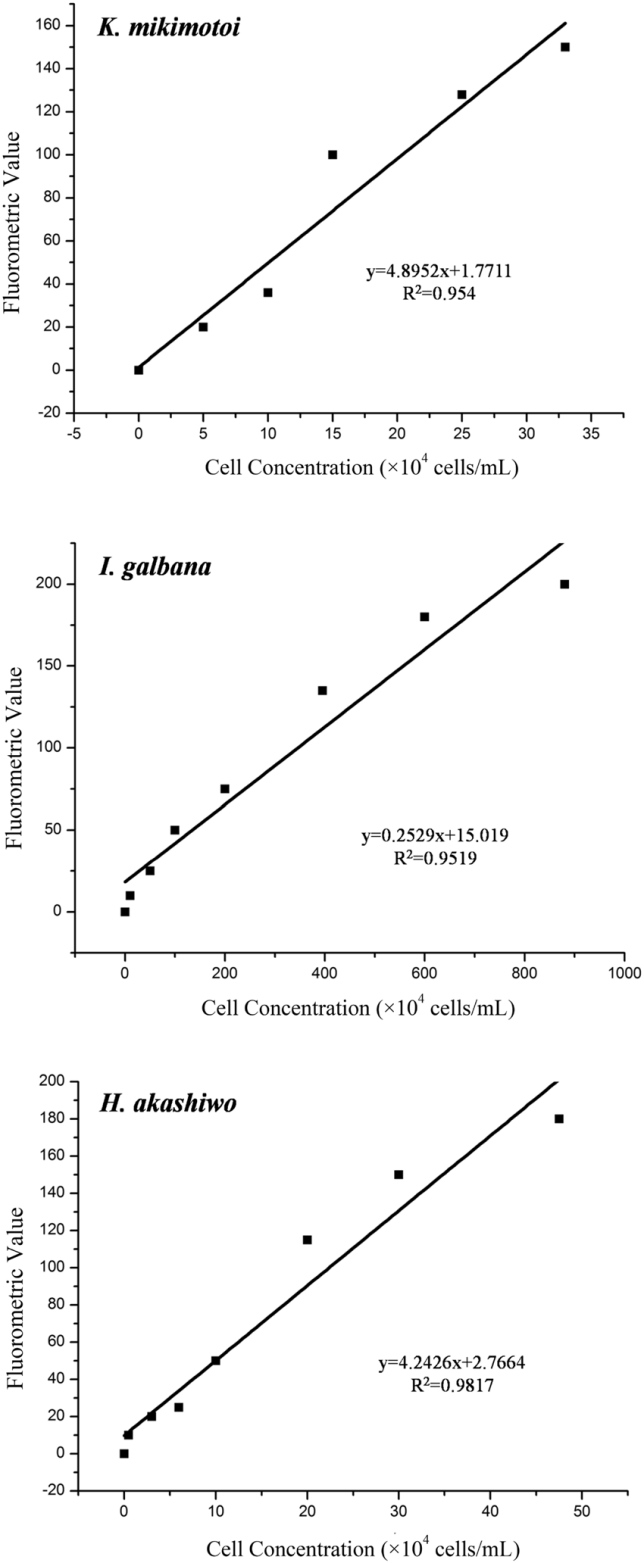
Regression line of microalgal cell concentration and fluorometric values.

In order to define an appropriate measurement on microalgal growth status, we investigated the chlorophyll fluorescence assay. It is well known that the biomass of microalgae, *in vivo* chlorophyll *a* (Chl *a*) fluorescence has been widely used and the Chl *a* fluorescence values can be measured accurately, fast, and repeatedly in microplates using microplate fluorometers^3,47^. In our experiments, the measurement for Chl *a* fluorometric values was selected at the excitation wavelength of 485 nm and the emission wavelength of 680 nm. Nevertheless, when microalgal cell death occurs, the light absorption properties and Chl *a* fluorescence yield of their pigment antenna are modified resulting in fluorescence quenching^47–49^. It has been reported that a good linear relationship was observed between the Chl *a* concentration and intensity of chlorophyll fluorescence when the Chl *a* concentration was within the range of 0.1 μg/mL to 5.0 μg/mL^26^. Consequently, we investigated the correlations between microalgal cellular concentrations and fluorometric values. Based on the regression analyses, it could be found that the microalgal cellular concentrations and fluorometric values of the three microalgae exhibited linear relationships, respectively (Fig. 3). According to the microalgal cellular concentration at 96 h (Fig. 2), it could be found that the observed Chl *a* fluorescence values and the corresponding microalgal cellular densities were in the linear range. The above analysis spoke well for the feasibility of using automated fluorometric values to quantify the microalgal growth. Ultimately, we determined to quantify the microalgal growth through measuring the chlorophyll fluorescence values by a microplate reader. Furthermore, the observed endpoint was determined at 96 h according to the growth curves of the tested microalgae (Fig. 2) and the linear regression analyses between microalgal cellular concentrations and fluorometric values (Fig. 3). It should be pointed that considerable test deviations occurred when we calculated the microalgal growth inhibition rates at the time points of 48 h and 72 h.

By repeated experiments and observations based on the above consideration, a microalgal growth inhibition microscale model was finally established. It should be noted that the previous reported microalgal microscale models were aimed at detecting environmental toxic samples as well as pollution chemicals^26,32^. Differing from the previous reports, we modified the microalgal microscale model with optimized conditions in the aspects of target marine microalgae, microalgal tested period, cosolvent, excitation wavelength, and calculation method. Therefore, the established model is specifically suitable for the assessment of the chemoecological effects of marine natural products with small molecular and micro- or trace quantity from marine organisms.

### The positive control and method validation

**Figure 4:**
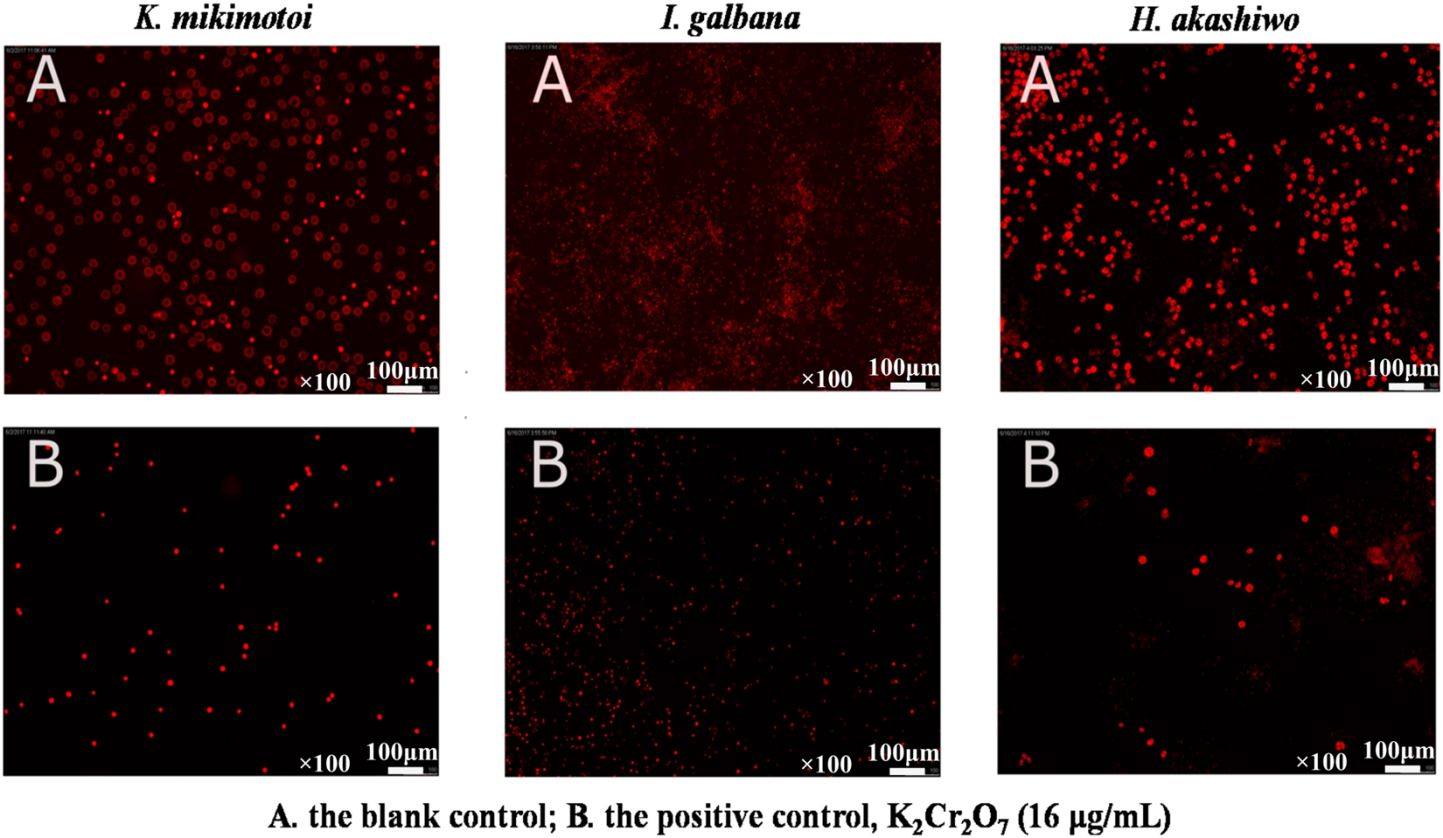
Fluorometric expression figure of the blank control and the positive control to qualitatively compare the biomass differences of three microalgae at 96 h observed by using inverted fluorescence microscope.

In our microalgal growth inhibition model, K_2_Cr_2_O_7_ was used as a positive control. To the best of our knowledge, there have not been any data available for the 96h-EC_50_ value of the K_2_Cr_2_O_7_ towards the three microalgae, *K. mikimotoi, I. galbana, and H. akashiwo* in microplates. These microalgae were widely used in tests in many fields, but there has been no report to evaluate their growth inhibition activities tested in Erlenmeyer flasks or microplates by using K_2_Cr_2_O_7_ as a positive control. For example, *I. galbana* was extensively used in testing the ecotoxicity of products such as petroleum in water, linear alkylbenzene sulfonate (LAS) and mental contaminant^11,27,34^. Whereas we noticed that K_2_Cr_2_O_7_ was used as a tested compound to evaluate the ecotoxicity towards other algal species, such as to *Desmodesmus subspicatus* with an EC_50_ value of 0.67‒0.80 µg/mL in 96-well microplate^32^. In the present study, the 96h-EC_50_ values were measured in 96-well microplate by fluorometric tests. The 96h-EC_50_ values of the positive control K_2_Cr_2_O_7_ towards the three microalgae, *K. mikimotoi, I. galbana*, and *H. akashiwo* were tested as 4.90‒5.29, 4.79‒5.24, 3.72‒4.02 µg/mL, respectively (Table 1). The reproducibility and feasibility of the established microplate microalgal biotest model were investigated. It was found that the relative standard deviation (RSD) of 96h-EC_50_ values of the positive control were 3.86% for *K. mikimotoi*, 4.51% for *I. galbana*, and 4.07% for *H. akashiwo*, respectively (Table 1), suggesting that the established model was feasible and stable. Specifically, the differences in microalgal biomass could be visually and qualitatively described with the aid of inverted fluorescence microscope. It could be clearly observed that the growth of three microalgal species was inhibited by K_2_Cr_2_O_7_, which is reflected in the cell viability and fluorescence intensity (Fig. 4).

**Table 1:** The stability of the microalgal microscale inhibition model validated by the positive control, K_2_Cr_2_O_7_.

Tested times	96h-EC_50_ (μg/mL)		
	*K. mikimotoi*	*I. galbana*	*H. akashiwo*
1	4.90 ± 0.29	4.79 ± 0.41	3.72 ± 0.32
2	5.29 ± 0.15	5.07 ± 0.37	3.96 ± 0.17
3	5.06 ± 0.31	5.24 ± 0.24	4.02 ± 0.26
RSD	3.86%	4.51%	4.07%

### Application of microalgal growth inhibition microscale model to marine natural products

The established model was applied to test the microalgal growth inhibition activities of the extracts, fractions, and scendary metabolites from soft corals, gorgonians, sponges and their symbiotic microorganisms collected from the South China Sea. As for *Alternaria* sp., a soft coral-derived fungus, the ethyl acetate extract of its fermentation broth showed inhibitory activity to *K. mikimotoi* with a growth inhibition rate of 76% at 50 μg/mL. The extract (30.2 g) was subjected to silica gel column chromatography to yield nine fractions (Fr. 1−Fr. 9). Two fractions, Fr. 6 and Fr. 7, were found to display microalgal growth inhibitory activities. Fr.6 exhibited the activity with the inhibition rates of 56% and 32% at the concentrations of 50 μg/mL and 10 μg/mL respectively, while Fr.7 with 96% and 84% at the same concentrations. Through further bioassay-guided isolation, two active compounds, tetrahydroaltersolanol E (6.0 mg, from Fr. 6) and altersolanol C (60 mg, from Fr.7), were obtained. By the same approaches as described for the soft coral-derived fungus *Alternaria* sp., other compounds were also isolated from corals, gorgonians, sponges and their symbiotic microorganisms under the guidance of microalgal growth inhibition model.

**Figure 5:**
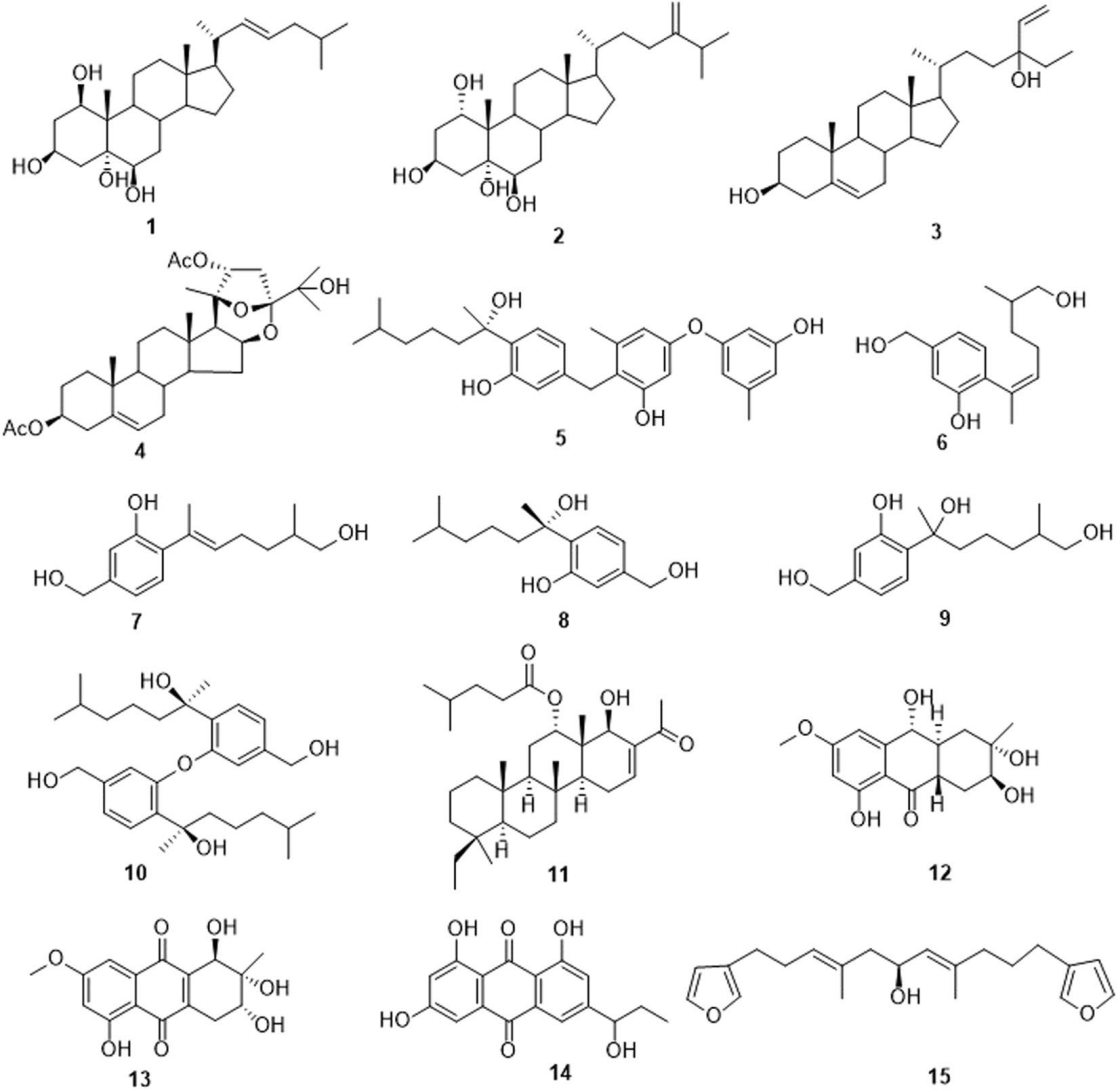
Structures of compounds 1–15.

All of the isolated secondary metabolites were tested for their microalgal growth inhibition activities with the established microalgal microscale model. Preliminary screening of 116 isolated compounds from marine organisms resulted in the discovery of 15 active compounds, including four steroids (**1**‒**4**), six bisabolane sesquiterpenoids (**5**‒**10**), one scalarane sesterterpene (**11**), three anthraquinoids (**12**‒**14**) and one difurano-sesterterpene (**15**) (Fig. 5). These compounds were characterized by comprehensive spectroscopic data and identified as follows: muristeroid G (**1**)^50^ was isolated from gorgonian *Anthogorgia caerulea*; numersterol A (**2**)^51^ from soft coral *Sinularia* sp.; saringosterol (**3**)^52^ and suberoretisteroid C (**4**)^3^ from gorgonian *Dichotella gemmacea*; expansol B (**5**)^53^, anhydrowaraterpols A and B (**6** and **7**)^54^, (S)-(+)-sydonol (**8**)^40^, waraterpol (**9**)^54^ and disydonol B (**10**)^40^ from sponge-derived fungus *Aspergillus* sp.; cateriofenone A (**11**)^41^ from sponge *Carteriospongia foliascens*; tetrahydroaltersolanol E (**12**)^42^ and altersolanol C (**13**)^42^ from soft coral-derived fungus *Alternaria* sp.; rhodoptilometrin (**14**)^55^ and 12,13-didehydrofurospongin-1 (**15**)^56^ from sponge *C. foliascens* (see Supplementary Table S1). All of these active compounds exhibited growth inhibition activity against at least one of three marine microalgal species (Table 2). Specifically, altersolanol C (**13**) demonstrated potent activity against *K. mikimotoi* with the 96h-EC_50_ value of 1.16 µg/mL, which was more than 4 times stronger than that of the positive control K_2_Cr_2_O_7_ for *K. mikimotoi*. Eight compounds (**1**‒**5**, **7**, **8**, **14**) showed more significant impact than other compounds on *I. galbana* with the 96h-EC_50_ values lower than or comparable to that of K_2_Cr_2_O_7_. For *H. akashiwo*, ten compounds (**2**, **4**‒**7**, **9**‒**11**, **14**, **15**) exhibited strong inhibitory activities with the 96h-EC_50_ values ranging from 2.74 to 10.3 µg/mL. Among these ten compounds, Expansol B (**5**) was the most powerful inhibitor against *H. akashiwo* with a EC_50_ value of 2.74 µg/mL, stronger than that of the positive control K_2_Cr_2_O_7_ (3.72 µg/mL) (Table 2). The above results demonstrated that different microalgal species displayed different sensitivities to the same compounds. Herein, it should be mentioned that some compounds were also found to be active on other chemoecological models. For instance, saringosterol (**3**) also showed lethal activity towards brine shrimp *Artemia salina*^40^, suberoretisteroid C (**4**) displayed antifouling activity against the larval settlement of barnacle *Balanus amphitrite*^3^, and suberoretisteroid C (**4**) and altersolanol C (**13**) exhibited ichthyotoxicity on the embryo of zebrafish *Danio rerio*^5^.

**Table 2:** Microalgal growth inhibition activity of compounds 1‒15 against microalgae (96h-EC_50_ values, μg/mL, *n* = 3).

Compd.	K. mikimotoi	I. galbana	H. akashiwo
1	>50.0	8.46 ± 0.37	12.2 ± 0.41
2	26.5 ± 0.32	4.26 ± 0.46	9.11 ± 0.66
3	>50.0	7.79 ± 0.36	33.7 ± 0.65
4	19.7 ± 0.24	3.09 ± 0.13	10.3 ± 0.33
5	—	10.7 ± 0.36	2.74 ± 0.20
6	—	31.8 ± 0.74	6.88 ± 0.58
7	—	2.15 ± 0.20	5.08 ± 0.19
8	—	10.8 ± 0.21	11.8 ± 0.22
9	—	>50.0	4.87 ± 0.21
10	—	>50.0	6.74 ± 0.34
11	—	>50.0	6.02 ± 0.35
12	42.3 ± 0.56	—	—
13	1.16 ± 0.11	—	—
14	ND	2.29 ± 0.41	6.91 ± 0.26
15	ND	33.6 ± 0.65	5.07 ± 0.37
K_2_Cr_2_O_7_^a^	4.90 ± 0.29	4.79 ± 0.41	3.72 ± 0.32

Note: a, positive control with K_2_Cr_2_O_7_, 2 µg/mL; “—”, had no activity; “ND”: not detected.

The above results revealed that the established model requires only very small amounts of the tested compounds, which is efficient for saving time and space. This model is suitable for the assessment of marine microalgal growth-inhibitory activity of marine natural products with micro- or trace quantity and could also be used in the bioactive screening and bioassay-guided isolation for the extracts and fractions from marine organisms.

## Conclusion

In this study, a convenient and operable microalgal growth inhibition microscale model was established by modifying the microalgal microscale model with optimized conditions involving in target marine microalgae, microalgal tested period, cosolvent, excitation wavelength for fluorescence observation, and calculation method. The microaglal growth inhibition activity was tested by using marine microalgae, 96-well microplate, together with fluorescence measurement to quantify microaglal biomass. The practicality and feasibility of the established model were validated by application of this model to the evaluation of microalgal growth inhibition activity of marine natural products derived from marine organisms. Fifteen compounds isolated from soft corals, gorgonians, sponges and their symbiotic microorganisms collected from the South China Sea were found to display microalgal growth inhibition activity. In conclusion, this method could be applied to evaluation of microaglal growth inhibition activity of marine natural products. Admittedly, this method could also be applicable for a range of toxicant testing. It could be prospected that more abundant marine natural products with microalgal growth-inhibitory activities would be discovered by using this model, facilitating in-depth understanding the chemoecological effects of marine chemical defensive substances.

## Electronic supplementary material

Supplementary Information
