# Supplementary material for: Microalgal Microscale Model for Microalgal Growth Inhibition Evaluation of Marine Natural Products
Source: Sci Rep. 2018 Jul 12;8:10541. doi: 10.1038/s41598-018-28980-z (PMC6043507; doi:10.1038/s41598-018-28980-z)

Supplementary Information

Microalgal Microscale Model for Microalgal Growth Inhibition Evaluation of Marine Natural Products

Qing Zhao[1,2,†], An-Na Chen[1,2,†], Shun-Xin Hu[3], Qian Liu[3], Min Chen[1,4], Lu Liu[1,2], Chang-Lun Shao[1,2,], Xue-Xi Tang[3,*], Chang-Yun Wang[1,2,5,*]*

1. Key Laboratory of Marine Drugs, the Ministry of Education of China, School of Medicine and Pharmacy, Ocean University of China, Qingdao 266003, China

2. Laboratory for Marine Drugs and Bioproducts, Qingdao National Laboratory for Marine Science and Technology, Qingdao 266071, P. R. China

3. College of Marine Life Sciences, Ocean University of China, Qingdao 266003, China

4. Marine Science & Technology Institute, College of Environmental Science & Engineering, Yangzhou University, 196#, Huayang West Street, Yangzhou 225127, China

5. Institute of Evolution & Marine Biodiversity, Ocean University of China, Qingdao 266003, China

† These authors contributed equally to this work.

* Corresponding author. Tel.: +86 532 82031536, e-mail: changyun@ouc.edu.cn (Chang-Yun Wang); Tel.: +86 532 82031381, e-mail: shaochanglun@ouc.edu.cn (Chang-Lun Shao); Tel.: +86 532 82032952, e-mail: tangxx@ouc.edu.cn (Xue-Xi Tang)

Supplementary Table S1 The detailed information of the active compounds

No.	Name	Source	Structure
**1**	muristeroid G	gorgonian *Anthogorgia caerulea*	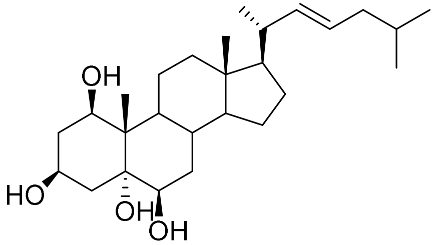
**2**	numersterol A	soft coral *Sinularia* sp.	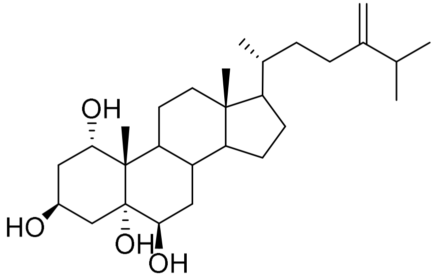
**3**	saringosterol	gorgonian *Dichotella gemmacea*	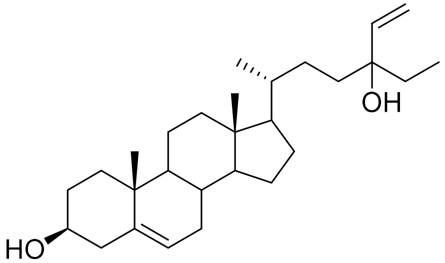

**4**	suberoretisteroid C	gorgonian *Dichotella gemmacea*	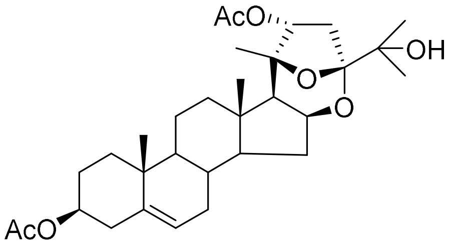
**5**	expansol B	sponge-derived fungus *Aspergillus* sp.	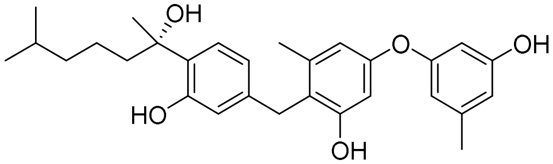
**6**	anhydrowaraterpols A	sponge-derived fungus *Aspergillus* sp.	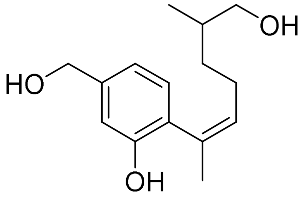
**7**	anhydrowaraterpols B	sponge-derived fungus *Aspergillus* sp.	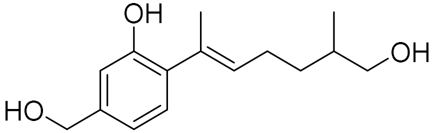
**8**	(S)-(+)-sydonol	sponge-derived fungus *Aspergillus* sp.	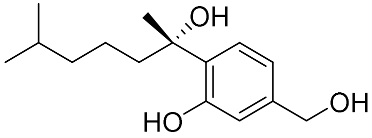

**9**	waraterpol	sponge-derived fungus *Aspergillus* sp.	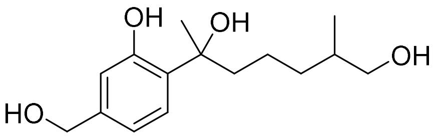
**10**	disydonol B	sponge-derived fungus *Aspergillus* sp.	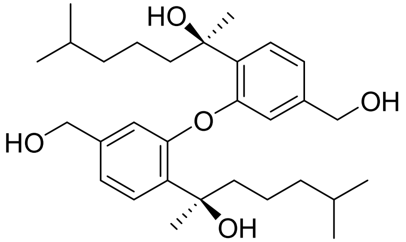
**11**	cateriofenone A	sponge *Carteriospongia foliascens*	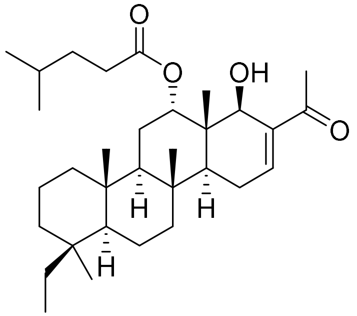
**12**	tetrahydroaltersolanol E	soft coral-derived fungus *Alternaria* sp.	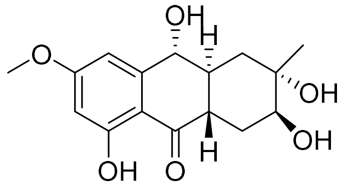

**13**	altersolanol C	soft coral-derived fungus *Alternaria* sp.	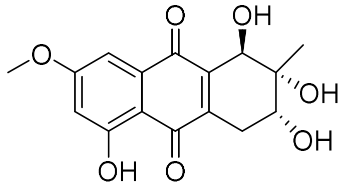
**14**	rhodoptilometrin	sponge *C. foliascens*	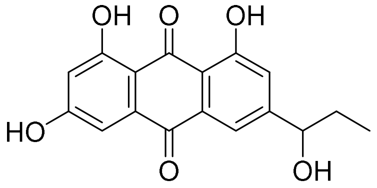
**15**	12,13-didehydrofurospongin-1	sponge *C. foliascens*	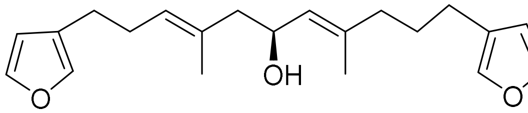

Supplementary Table S2 Fluorometric values under different concentrations and EC_{50} values (μg/mL) of the active compounds

K. mikimotoi																			
**No.**	**50μg/mL**			**10μg/mL**			**2μg/mL**			**0.4μg/mL**			**0.08μg/mL**			**0.00μg/mL**			**EC_{50}**
**1**	25.785	22.674	25.429	31.736	27.937	28.665	35.933	37.827	37.118	43.276	39.332	38.828	41.302	40.936	42.557	41.47	41.113	42.656	>50.0
**2**	13.227	11.982	13.789	17.129	19.274	17.082	24.862	27.841	23.219	37.282	36.774	39.316	43.982	40.862	40.793	39.274	43.031	44.277	26.5±0.32
**3**	26.939	25.378	23.142	31.289	34.274	35.591	36.947	42.478	37.701	40.553	42.386	40.316	46.229	42.486	43.864	46.375	40.664	46.393	>50.0
**4**	5.372	6.182	5.91	17.395	19.967	14.72	30.563	28.763	28.687	36.183	38.642	37.968	41.294	39.566	38.138	37.502	43.696	44.04	19.7±0.24
**5**	–	–	–	–	–	–	–	–	–	–	–	–	–	–	–	–	–	–	–
**6**	–	–	–	–	–	–	–	–	–	–	–	–	–	–	–	–	–	–	–
**7**	–	–	–	–	–	–	–	–	–	–	–	–	–	–	–	–	–	–	–
**8**	–	–	–	–	–	–	–	–	–	–	–	–	–	–	–	–	–	–	–
**9**	–	–	–	–	–	–	–	–	–	–	–	–	–	–	–	–	–	–	–
**10**	–	–	–	–	–	–	–	–	–	–	–	–	–	–	–	–	–	–	–
**11**	–	–	–	–	–	–	–	–	–	–	–	–	–	–	–	–	–	–	–
**12**	17.836	19.852	16.357	30.684	29.871	33.199	38.725	36.086	35.927	37.258	39.074	37.146	40.823	38.26	40.638	44.528	39.571	41.901	42.3±0.56
**13**	0.895	1.184	0.818	10.264	8.395	5.948	14.384	13.622	12.503	22.407	20.841	24.177	34.371	30.508	31.252	44.528	39.571	41.901	1.16±0.11
**14**	–	–	–	–	–	–	–	–	–	–	–	–	–	–	–	–	–	–	–
**15**	–	–	–	–	–	–	–	–	–	–	–	–	–	–	–	–	–	–	–
I. galbana																			

No.	50μg/mL			10μg/mL			2μg/mL			0.4μg/mL			0.08μg/mL			0.00μg/mL			EC_{50}
**1**	2.354	3.872	3.745	34.27	33.481	35.058	55.382	52.094	53.909	70.371	74.948	84.431	101.20	98.329	90.886	106.35	111.20	106.37	8.46±0.37
**2**	2.993	3.062	5.028	21.841	20.072	18.287	37.841	40.072	32.765	68.359	69.735	74.462	106.74	96.083	94.927	109.38	115.37	107.35	4.26±0.46
**3**	10.371	6.984	9.196	27.618	30.263	24.271	45.073	49.619	48.184	79.036	74.71	71.224	101.40	96.528	98.971	114.39	117.37	103.84	7.79±0.36
**4**	0.736	1.285	1.838	17.305	15.009	15.095	42.831	37.946	39.056	64.037	67.825	71.834	82.518	90.472	90.803	117.39	103.57	99.742	3.09±0.13
**5**	3.029	7.367	6.488	30.163	29.452	24.457	63.073	67.251	65.68	76.937	75.205	83.406	85.062	90.273	91.007	117.36	109.47	105.93	10.7±0.36
**6**	41.285	36.961	34.674	59.836	57.033	62.762	79.371	83.615	80.533	102.58	96.487	91.895	118.24	105.83	106.82	117.48	109.83	104.73	31.8±0.74
**7**	5.375	8.092	7.859	13.461	16.382	12.291	40.217	35.033	30.362	66.419	72.174	78.794	88.372	90.379	100.18	117.48	109.39	114.81	2.15±0.20
**8**	11.338	13.294	14.532	33.596	30.148	34.897	59.367	53.284	57.696	85.337	80.362	83.543	107.36	103.47	96.938	106.78	117.85	120.26	10.8±0.21
**9**	65.274	59.392	65.012	81.359	76.372	77.183	88.366	93.217	95.767	102.48	108.36	94.489	112.49	107.37	100.09	117.36	120.41	109.38	>50.0
**10**	66.381	71.903	75.179	82.307	75.164	73.744	81.266	76.284	87.11	98.357	89.351	96.577	106.36	110.38	96.771	109.37	116.10	120.48	>50.0
**11**	78.372	82.395	74.938	102.048	106.48	95.889	116.38	120.47	107.26	119.37	109.37	123.35	123.48	109.68	121.44	123.22	113.34	118.48	>50.0
**12**	–	–	–	–	–	–	–	–	–	–	–	–	–	–	–	–	–	–	–
**13**	–	–	–	–	–	–	–	–	–	–	–	–	–	–	–	–	–	–	–
**14**	5.429	3.958	5.034	19.371	18.349	17.968	40.327	33.516	35.819	71.364	68.306	70.129	92.317	87.358	90.469	118.32	116.36	105.27	2.29±0.41
**15**	36.545	33.093	31.289	88.037	80.538	83.388	99.734	92.851	94.199	106.38	112.84	102.04	118.30	108.38	118.59	120.36	119.36	108.48	33.6±0.65
H. akashiwo																			
**No.**	**50μg/mL**			**10μg/mL**			**2μg/mL**			**0.4μg/mL**			**0.08μg/mL**			**0.00μg/mL**			**EC_{50}**
**1**	5.638	7.462	8.698	23.471	19.364	19.019	35.618	32.584	33.181	48.361	48.572	40.069	50.639	60.375	53.481	66.473	63.092	58.274	12.2±0.41
**2**	4.327	2.583	4.438	17.365	14.057	19.382	36.935	32.824	35.298	42.721	48.824	45.843	56.951	48.087	48.072	59.426	64.073	64.078	9.11±0.66

**3**	23.368	22.413	18.557	40.026	35.417	33.785	43.084	35.742	38.488	48.851	50.742	43.931	56.957	48.164	56.189	57.367	55.382	61.425	33.7±0.65
**4**	4.582	7.439	6.079	21.648	18.376	15.767	33.652	34.572	29.692	46.517	42.489	42.066	47.842	52.529	51.108	57.357	59.539	66.394	10.3±0.33
**5**	4.577	4.638	6.332	17.492	14.036	14.984	20.947	30.583	20.624	33.738	31.725	28.634	45.301	40.267	37.815	57.385	58.382	56.497	2.74±0.20
**6**	6.395	5.094	3.99	19.482	20.307	16.766	31.591	27.462	28.421	37.592	36.406	33.745	51.307	47.583	43.131	64.395	60.187	56.073	6.88±0.58
**7**	4.875	6.963	7.103	21.856	19.883	17.214	26.926	21.573	23.335	42.742	38.005	37.75	53.082	50.995	48.424	64.562	67.834	61.857	5.08±0.19
**8**	6.852	8.624	5.593	13.847	15.093	13.135	32.857	28.461	30.093	48.403	43.591	43.52	54.965	55.317	54.494	56.57	58.384	61.965	11.8±0.22
**9**	6.487	8.435	7.917	16.344	13.462	12.397	25.737	22.645	21.107	42.583	37.476	36.949	48.683	50.475	45.145	56.265	60.468	66.379	4.87±0.21
**10**	5.489	5.372	4.752	15.384	21.385	19.104	28.471	22.469	31.943	38.837	36.623	37.656	48.387	42.397	49.427	63.978	57.326	59.322	6.74±0.34
**11**	1.385	2.563	4.229	18.372	17.045	15.528	30.271	27.049	23.693	33.872	39.964	36.223	48.301	42.496	49.411	63.295	58.391	54.395	6.02±0.35
**12**	–	–	–	–	–	–	–	–	–	–	–	–	–	–	–	–	–	–	–
**13**	–	–	–	–	–	–	–	–	–	–	–	–	–	–	–	–	–	–	–
**14**	7.383	6.256	5.148	20.613	18.958	14.924	26.964	24.563	23.752	38.052	33.726	39.433	51.736	57.973	57.207	63.594	66.392	58.375	6.91±0.26
**15**	6.974	9.386	9.285	22.746	18.573	19.011	26.734	30.025	24.33	32.856	27.953	34.57	46.385	51.562	48.468	65.082	57.995	63.424	5.07±0.37

Supplementary Fig. S1 The growth process of three microalgae during the 30-d period in 250mL Erlenmeyer flasks (*n*=3)

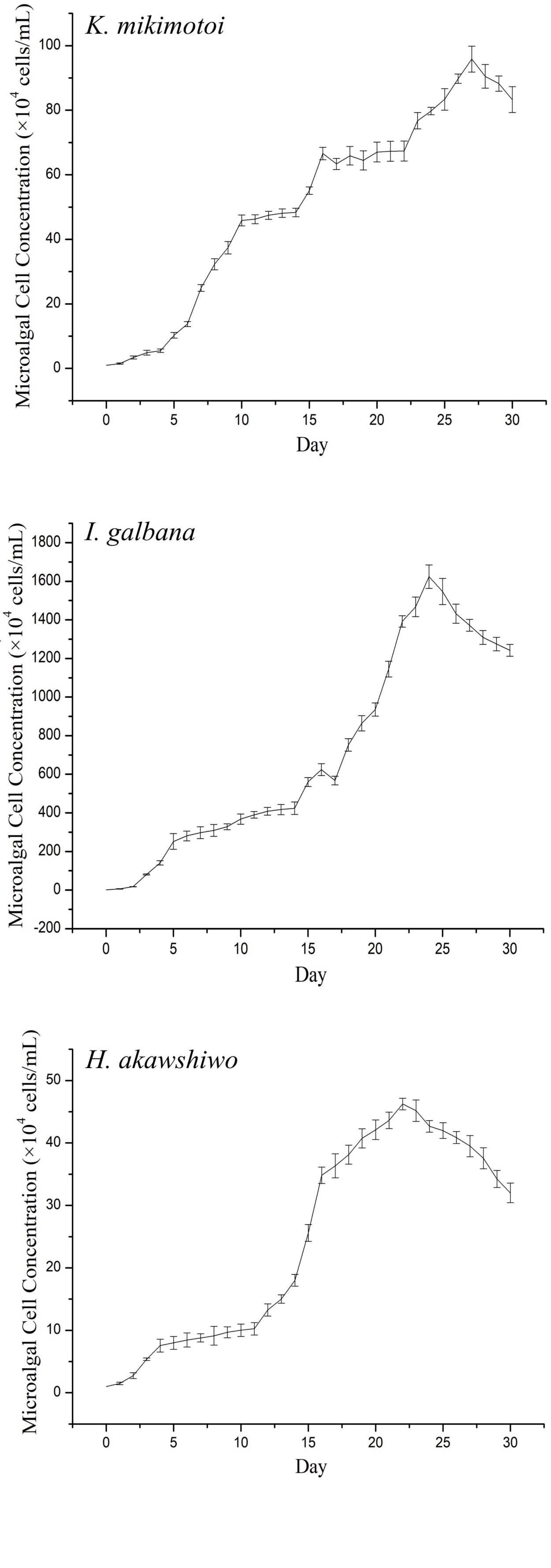

Supplement: Supplementary file 1 — Supplementary Information [file 41598_2018_28980_MOESM1_ESM.pdf]
